# Supplementary material for: Exploring the Diversity and Ecological Dynamics of Palm Leaf Spotting Fungi—A Case Study on Ornamental Palms in Portugal
Source: J Fungi (Basel). 2025 Jan 7;11(1):43. doi: 10.3390/jof11010043 (PMC11766901; doi:10.3390/jof11010043)
Supplement: Supplementary file 1 [file jof-11-00043-s001.zip › jof-3401154-supplementary.pdf]

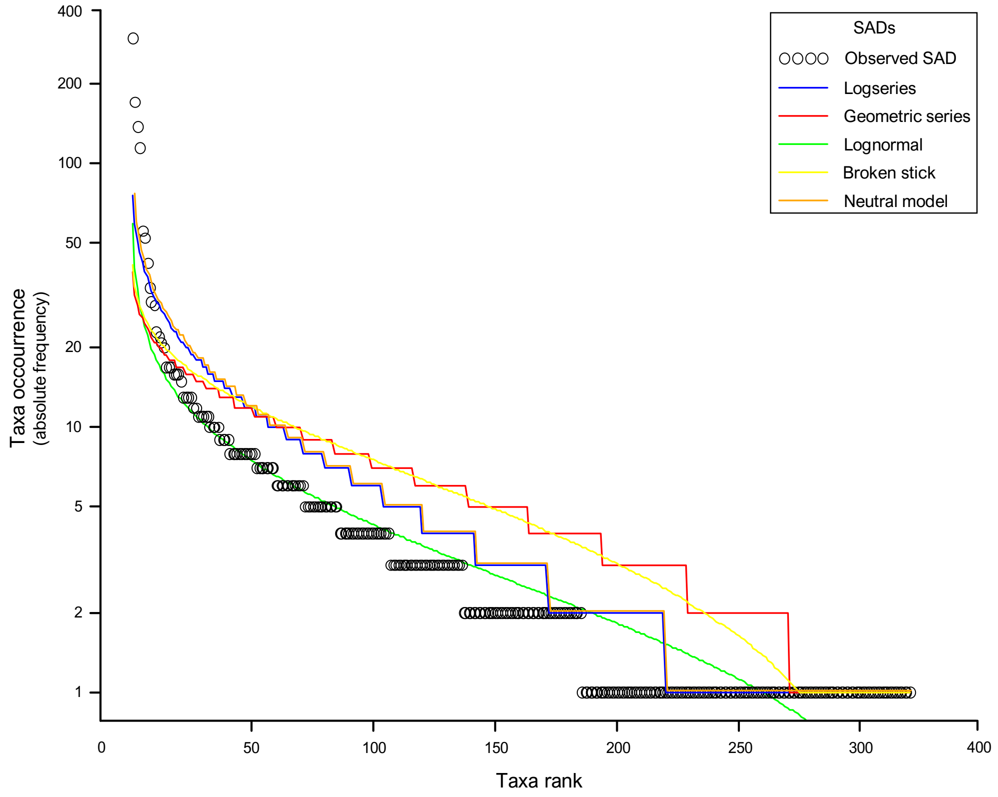

Figure S1. Observed and modelled abundance distributions for assemblages of palm leaf spotting fungi (PLSF). The y-axis represents the observed or expected absolute occurrence of each taxon. Each taxon is ranked from the most to least abundant along the x-axis. Empty circles represent observed values; solid lines represent expected values according to the species abundance distribution (SAD) model fitted and are referred to in the chart legend.

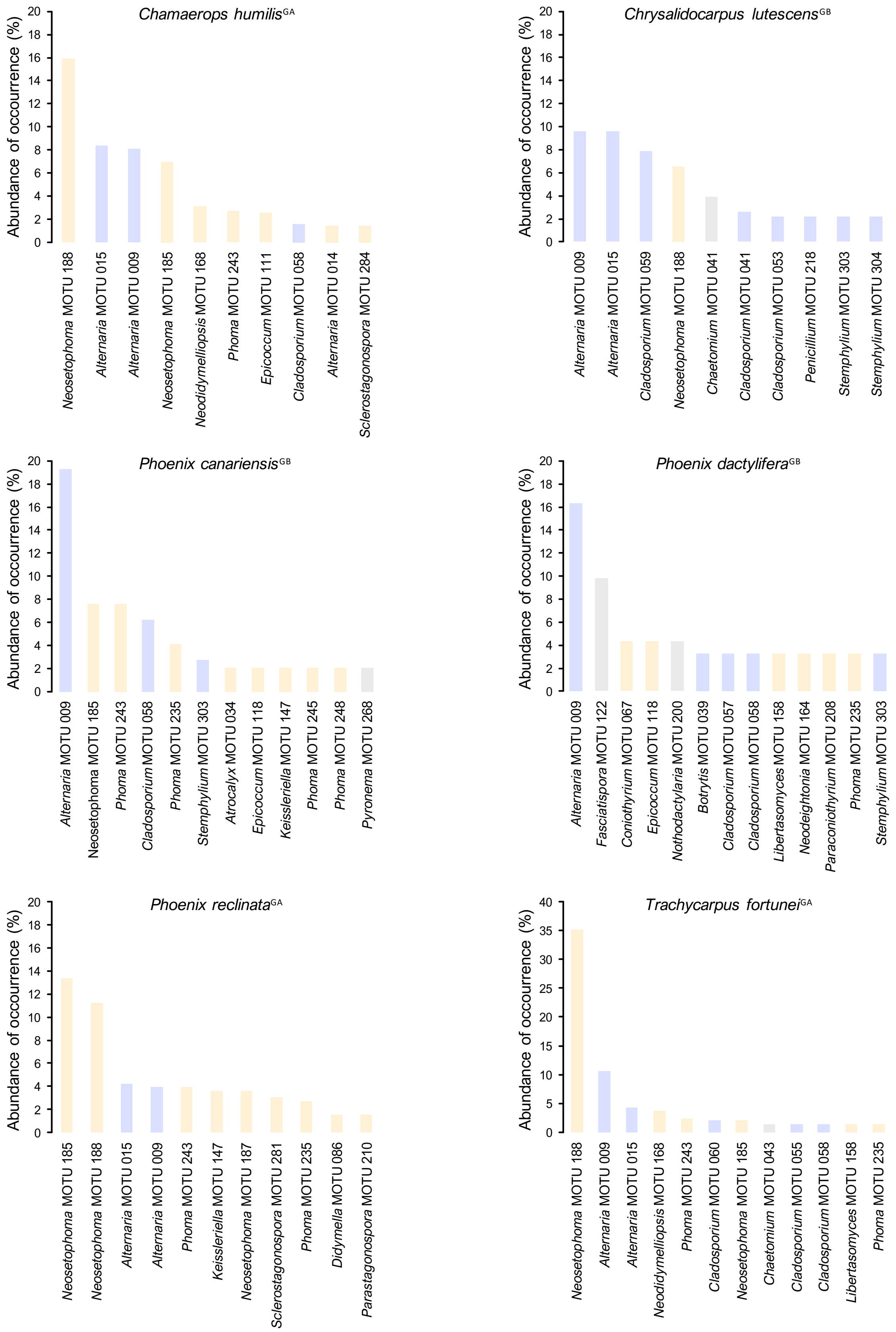

Figure S2. Top ten molecular operational taxonomic units (MOTU) by assemblage type and host species. Each panel depicts the percentage abundance of occurrence (AO) of the ten most documented MOTUs found associated with foliar lesions by host species. In some palm host species, more than ten MOTUs are shown, as the tenth most documented MOTU exhibited the same AO in more than one MOTU. Colours are according to assemblage types and are referred to in the chart legend. The

groups defined according to trends in fungal biodiversity are identified by superscript abbreviations as group A (^{GA}) and group B (^{GB}) in the respective palm host species. It should be noted that the scale of the axis in the panel of *Trachycarpus fortunei* has been modified to allow the representation of the most abundant MOTU.

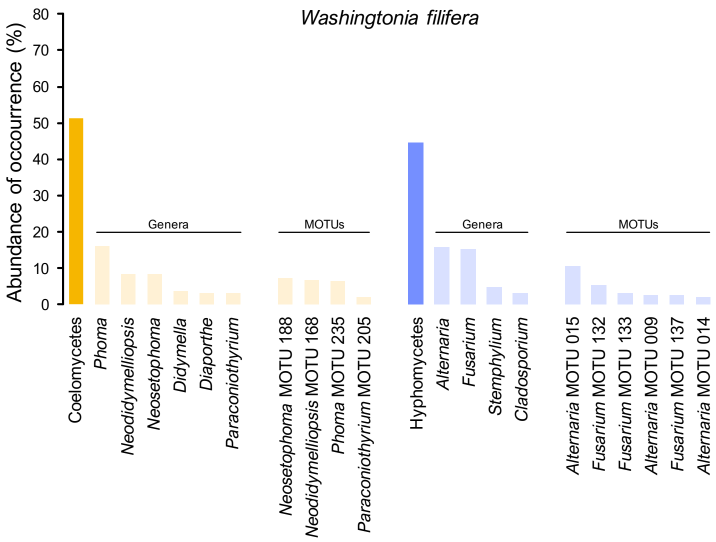

Figure S3. Top ten genera and molecular operational taxonomic units (MOTU) by assemblage type of *Washingtonia filifera*. The plot depicts the percentage abundance of occurrence (AO) of the ten most documented genera and MOTUs found associated with foliar lesions of *Washingtonia filifera*. Colours are according to assemblage types and are referred to in the first bar of each colour.

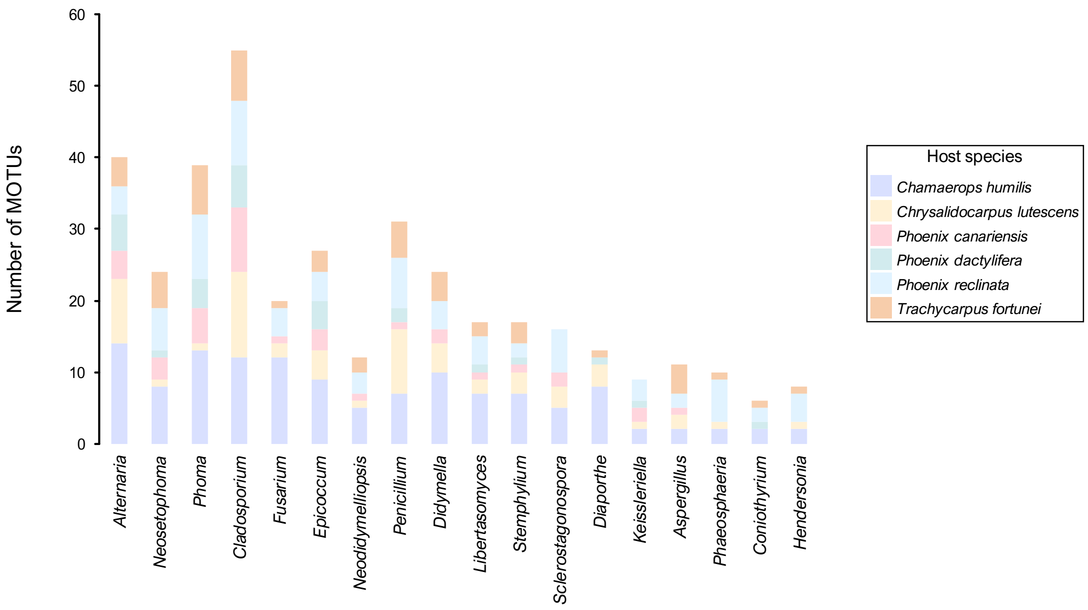

Figure S4. Genera recorded on at least four host species and corresponding number of molecular operational taxonomic units (MOTU). Genera with an abundance of occurrence (AO) ≤ 0.45 % were excluded. Colours are according to host species and are referred to in the chart legend.

Table S1. Distribution of molecular operational taxonomic units (MOTU) by frequency groups. MOTUs are grouped based on their abundance of occurrence as very frequent (> 5 %), frequent (> 2–5 %), infrequent (> 1–2 %) or rare (≤ 1 %).

Frequency group/MOTU[1]	Measures of population status[2]	
	AO (%)	FO (%)
Very frequent		
Neosetophoma MOTU 188	14.44	25.37
Alternaria MOTU 009	8.33	50.75
Alternaria MOTU 015	6.64	19.40
Neosetophoma MOTU 185	5.52	21.64
Frequent		
Phoma MOTU 243	2.71	28.36
Neodidymelliopsis MOTU 168	2.52	10.45
Phoma MOTU 235	2.03	17.91
Infrequent		
Cladosporium MOTU 058	1.65	20.90
Cladosporium MOTU 059	1.45	14.18
Epicoccum MOTU 111	1.41	7.46
Neosetophoma MOTU 187	1.11	9.70
Stemphylium MOTU 303	1.07	10.45
Fusarium MOTU 132	1.02	6.72
Rare		
Libertasomyces MOTU 158	0.97	11.94
Epicoccum MOTU 118	0.82	9.70
Keissleriella MOTU 147	0.82	5.97
Sclerostagonospora MOTU 281	0.82	5.97
Cladosporium MOTU 060	0.78	11.94
Penicillium MOTU 221	0.78	11.94
Alternaria MOTU 014	0.78	3.73
Sclerostagonospora MOTU 284	0.73	6.72
Phoma MOTU 245	0.63	7.46
Didymella MOTU 089	0.63	5.97
Libertasomyces MOTU 154	0.63	5.97
Fasciatispora MOTU 122	0.63	5.22
Cladosporium MOTU 055	0.58	8.21
Didymella MOTU 086	0.58	6.72
Cladosporium MOTU 056	0.53	8.21
Epicoccum MOTU 114	0.53	7.46

Stemphylium MOTU 306	0.53	5.97
Coniothyrium MOTU 067	0.53	5.22
Fusarium MOTU 137	0.53	4.48
Penicillium MOTU 217	0.48	7.46
Nothophoma MOTU 202	0.48	5.97
Chaetomium MOTU 041	0.48	5.22
Phoma MOTU 240	0.48	5.22
Pyronema MOTU 268	0.44	6.72
Botrytis MOTU 039	0.44	5.97
Sclerostagonospora MOTU 283	0.44	5.97
Alternaria MOTU 019	0.44	3.73
Aspergillus MOTU 027	0.39	5.22
Diaporthe MOTU 082	0.39	5.22
Cladosporium MOTU 053	0.39	4.48
Diaporthe MOTU 077	0.39	4.48
Penicillium MOTU 218	0.39	4.48
Phoma MOTU 247	0.39	4.48
Alternaria MOTU 008	0.39	3.73
Cladosporium MOTU 045	0.39	3.73
Hendersonia MOTU 144	0.39	3.73
Parastagonospora MOTU 210	0.39	2.99
Libertasomyces MOTU 156	0.39	1.49
Cladosporium MOTU 054	0.34	5.22
Phoma MOTU 249	0.34	5.22
Fusarium MOTU 139	0.34	4.48
Neodeightonia MOTU 164	0.34	4.48
Stemphylium MOTU 304	0.34	4.48
Fusarium MOTU 125	0.34	2.99
Fusarium MOTU 133	0.34	2.99
Hendersonia MOTU 145	0.34	2.99
Aspergillus MOTU 028	0.29	4.48
Didymella MOTU 090	0.29	4.48
Keissleriella MOTU 148	0.29	4.48
Phaeosphaeria MOTU 224	0.29	4.48
Cladosporium MOTU 049	0.29	3.73
Phoma MOTU 239	0.29	3.73
Didymocyrtis MOTU 098	0.29	2.99

Phoma MOTU 246	0.29	2.99
Pithomyces MOTU 254	0.29	2.99
Rhizopus MOTU 270	0.29	2.99
Didymella MOTU 091	0.29	2.24
Nigrospora MOTU 195	0.29	2.24
Cladosporium MOTU 051	0.24	3.73
Diaporthe MOTU 075	0.24	3.73
Fusarium MOTU 131	0.24	3.73
Stagonosporopsis MOTU 298	0.24	3.73
Allophoma MOTU 003	0.24	2.99
Atrocalyx MOTU 034	0.24	2.99
Chaetomium MOTU 040	0.24	2.99
Didymella MOTU 088	0.24	2.99
Libertasomyces MOTU 155	0.24	2.99
Neodidymelliopsis MOTU 166	0.24	2.99
Nothodactylaria MOTU 200	0.24	2.99
Chaetomium MOTU 043	0.24	2.24
Colletotrichum MOTU 062	0.24	2.24
Fusarium MOTU 138	0.24	2.24
Alternaria MOTU 010	0.19	2.99
Alternaria MOTU 012	0.19	2.99
Aspergillus MOTU 030	0.19	2.99
Cladosporium MOTU 048	0.19	2.99
Cladosporium MOTU 057	0.19	2.99
Keissleriella MOTU 146	0.19	2.99
Neodidymelliopsis MOTU 169	0.19	2.99
Neofusicoccum MOTU 174	0.19	2.99
Neosetophoma MOTU 190	0.19	2.99
Penicillium MOTU 211	0.19	2.99
Penicillium MOTU 214	0.19	2.99
Penicillium MOTU 216	0.19	2.99
Penicillium MOTU 219	0.19	2.99
Phoma MOTU 234	0.19	2.99
Phoma MOTU 248	0.19	2.99
Stagonosporopsis MOTU 297	0.19	2.99
Neodeightonia MOTU 165	0.19	2.24
Sordaria MOTU 292	0.19	2.24

Epicoccum MOTU 113	0.19	1.49
Paraconiothyrium MOTU 208	0.19	1.49
Paraconiothyrium MOTU 205	0.19	0.75
Alternaria MOTU 017	0.15	2.24
Alternaria MOTU 018	0.15	2.24
Arthrinium MOTU 023	0.15	2.24
Diaporthe MOTU 081	0.15	2.24
Diatrypella MOTU 085	0.15	2.24
Didymella MOTU 094	0.15	2.24
Didymella MOTU 096	0.15	2.24
Dimorpha MOTU 105	0.15	2.24
Dothiorella MOTU 107	0.15	2.24
Epicoccum MOTU 116	0.15	2.24
Fusarium MOTU 126	0.15	2.24
Libertasomyces MOTU 157	0.15	2.24
Libertasomyces MOTU 159	0.15	2.24
Neofusicoccum MOTU 176	0.15	2.24
Paraconiothyrium MOTU 204	0.15	2.24
Phoma MOTU 250	0.15	2.24
Sclerostagonospora MOTU 286	0.15	2.24
Septoria MOTU 289	0.15	2.24
Stemphylium MOTU 299	0.15	2.24
Xenocylindrosporium MOTU 319	0.15	2.24
Didymella MOTU 093	0.15	1.49
Epicoccum MOTU 115	0.15	1.49
Neosetophoma MOTU 191	0.15	1.49
Nigrospora MOTU 196	0.15	1.49
Paraconiothyrium MOTU 206	0.15	1.49
Phaeosphaeria MOTU 226	0.15	1.49
Phaeosphaeria MOTU 227	0.15	1.49
Phoma MOTU 244	0.15	1.49
Sordaria MOTU 290	0.15	1.49
Fusarium MOTU 134	0.15	0.75
Rare (doubletons)	0.10	0.75 or 1.49
Acremonium MOTU 001, *Alternaria* MOTU 006*, MOTU 011*, MOTU 013* and MOTU 016*, *Arthrinium* MOTU 024*, *Aspergillus* MOTU 032*, *Atrocalyx* MOTU 033*, *Chaetomium* MOTU 042* and MOTU 044*, *Cladosporium* MOTU 050*, MOTU 046* and MOTU 061*, *Colletotrichum* MOTU 065*, *Coniochaeta* MOTU 066*, *Coniothyrium* MOTU 068*, *Didymella* MOTU 087*, *Didymocyrtis* MOTU 100*, *Ectophoma* MOTU 110*, *Epicoccum* MOTU 119, *Hendersonia* MOTU 142,		

Leptosphaerulina MOTU 152*, *Morinia* MOTU 162* and MOTU 163*, *Neodidymelliopsis* MOTU 167*, *Neofusicoccum* MOTU 175*, *Neopestalotiopsis* MOTU 178*, *Neosetophoma* MOTU 186*, *Neosulcatispora* MOTU 193*, *Paraconiothyrium* MOTU 207, *Penicillium* MOTU 212*, MOTU 220* and MOTU 222*, *Pithomyces* MOTU 252, MOTU 255* and MOTU 256, *Plenodomus* MOTU 257* and MOTU 258*, *Plicaria* MOTU 259*, *Preussia* MOTU 263*, *Pseudopithomyces* MOTU 266, *Sarocladium* MOTU 274*, *Sclerostagonospora* MOTU 282*, *Sordaria* MOTU 291 and MOTU 293, *Stemphylium* MOTU 301* and MOTU 305, *Trichoderma* MOTU 315*, *Xenocylindrosporium* MOTU 320*		
Rare (singletons)	0.05	0.75
Acremonium MOTU 002, *Alternaria* MOTU 004, MOTU 005, MOTU 007 and MOTU 020, *Antrodia* MOTU 021, *Aplosporella* MOTU 022, *Arthrinium* MOTU 025, *Ascochyta* MOTU 026, *Aspergillus* MOTU 029 and MOTU 031, *Bartalinia* MOTU 035, *Bipolaris* MOTU 036, *Blastobotrys* MOTU 037, *Botryosphaeria* MOTU 038, *Cladosporium* MOTU 047 and MOTU 052, *Colletotrichum* MOTU 063 and MOTU 064, *Coprinopsis* MOTU 069, *Coriolopsis* MOTU 070, *Cryptovalsa* MOTU 071, *Cylindrobasidium* MOTU 072, *Cytospora* MOTU 073, *Daldinia* MOTU 074, *Diaporthe* MOTU 078, MOTU 079, MOTU 080, MOTU 083, MOTU 084 and MOTU 076, *Didymella* MOTU 092, MOTU 095 and MOTU 097, *Didymocyrtis* MOTU 099, MOTU 101, MOTU 102 and MOTU 103, *Didymosphaeria* MOTU 104, *Diplodia* MOTU 106, *Dothiorella* MOTU 108 and MOTU 109, *Epicoccum* MOTU 112, MOTU 117, MOTU 120 and MOTU 121, *Foliophoma* MOTU 123 and MOTU 124, *Fusarium* MOTU 127, MOTU 128, MOTU 129, MOTU 130, MOTU 135, MOTU 136 and MOTU 140, *Harzia* MOTU 141, *Hendersonia* MOTU 143, *Lanzia* MOTU 149, *Lecanicillium* MOTU 150 and MOTU 151, *Leptosphaerulina* MOTU 153, *Libertasomyces* MOTU 160, *Monilia* MOTU 161, *Neodidymelliopsis* MOTU 170, MOTU 171 and MOTU 172, *Neoeutypella* MOTU 173, *Neofusicoccum* MOTU 177, *Neopestalotiopsis* MOTU 179 and MOTU 180, *Neosetophoma* MOTU 181, MOTU 182, MOTU 183, MOTU 189 and MOTU 184, *Neostagonospora* MOTU 192, *Neurospora* MOTU 194, *Nigrospora* MOTU 197, MOTU 198 and MOTU 199, *Nothodactylaria* MOTU 201, *Palmeiromyces* MOTU 203, *Paraphaeosphaeria* MOTU 209, *Penicillium* MOTU 215 and MOTU 213, *Peziza* MOTU 223, *Phaeosphaeria* MOTU 225, MOTU 228, MOTU 229 and MOTU 230, *Phanerochaete* MOTU 232 and MOTU 231, *Phlebia* MOTU 233, *Phoma* MOTU 237, MOTU 238, MOTU 241, MOTU 242 and MOTU 236, *Phylosticta* MOTU 251, *Pithomyces* MOTU 253, *Plicaria* MOTU 260, *Preussia* MOTU 262 and MOTU 261, *Pseudoconiothyrium* MOTU 264, *Pseudogymnoascus* MOTU 265, *Pseudopithomyces* MOTU 267, *Pyronema* MOTU 269, *Rhizopus* MOTU 271 and MOTU 272, *Sardiniella* MOTU 273, *Sarocladium* MOTU 275, MOTU 276 and MOTU 277, *Schizothecium* MOTU 278, *Sclerostagonospora* MOTU 280, MOTU 285 and MOTU 279, *Scopulariopsis* MOTU 288 and MOTU 287, *Sordaria* MOTU 294 and MOTU 295, *Stachybotrys* MOTU 296, *Stemphylium* MOTU 300, MOTU 302, MOTU 307, MOTU 308 and MOTU 309, *Tamaracicola* MOTU 310, *Thielavia* MOTU 311, *Trametes* MOTU 312, *Tricharina* MOTU 313 and MOTU 314, *Wallemia* MOTU 316 and MOTU 317, *Wojnowiciella* MOTU 318		

[1] Rare MOTUs represented by doubletons (two fungal records) or singletons (one fungal record) are listed in alphabetic order. Doubletons isolated from two samples (FO = 1.49 %) are noted by a superscript asterisk (*).

[2] AO: abundance of occurrence; FO: frequency of occurrence.

Note: genera are listed in descending order of AO.

Table S2. Percentage of total positive, negative, or random pairwise interactions for each genus involved in significant combinations and their respective number of counts.

Genus	Percentage (%) of pairwise interactions			Positive counts	Negative counts	Random counts
	Positive	Negative	Random[1]			
Acremonium	0.00	0.00	100.00	0	0	2
Allophoma	0.00	0.00	100.00	0	0	5
Alternaria	22.95	0.00	77.05	14	0	47
Arthrinium	12.50	0.00	87.5	1	0	7
Aspergillus	11.54	0.00	88.46	3	0	23
Atrocalyx	12.50	0.00	87.5	1	0	7
Botrytis	0.00	0.00	100.00	0	0	14
Chaetomium	0.00	0.00	100.00	0	0	15
Cladosporium	8.20	0.00	91.80	5	0	56
Colletotrichum	14.29	0.00	85.71	1	0	6
Coniochaeta	0.00	0.00	100.00	0	0	2
Coniothyrium	6.67	0.00	93.33	1	0	14
Diaporthe	42.31	0.00	57.69	11	0	15
Diatrypella	25.00	0.00	75.00	1	0	3
Didymella	58.62	0.00	41.38	17	0	12
Didymocyrtis	20.00	0.00	80.00	3	0	12
Dimorpha	0.00	0.00	100.00	0	0	4
Dothiorella	20.00	0.00	80.00	1	0	4
Ectophoma	0.00	0.00	100.00	0	0	2
Epicoccum	35.14	0.00	64.86	13	0	24
Fasciatispora	0.00	0.00	100.00	0	0	11
Foliophoma	0.00	0.00	100.00	0	0	2
Fusarium	58.62	0.00	41.38	17	0	12
Hendersonia	60.00	0.00	40.00	9	0	6
Keissleriella	33.33	0.00	66.67	7	0	14
Lecanicillium	0.00	0.00	100.00	0	0	2
Leptosphaerulina	25.00	0.00	75.00	1	0	3
Libertasomyces	37.93	0.00	62.07	11	0	18
Morinia	0.00	0.00	100.00	0	0	5
Neodeightonia	13.33	0.00	86.67	2	0	13
Neodidymelliopsis	57.69	0.00	42.31	15	0	11
Neofusicoccum	35.71	0.00	64.29	5	0	9
Neopestalotiopsis	0.00	0.00	100.00	0	0	5

Neosetophoma	36.73	0.00	63.27	18	0	31
Neosulcatispora	0.00	0.00	100.00	0	0	2
Nigrospora	0.00	0.00	100.00	0	0	14
Nothodactylaria	0.00	0.00	100.00	0	0	7
Nothophoma	50.00	0.00	50.00	7	0	7
Paraconiothyrium	0.00	0.00	100.00	0	0	8
Parastagonospora	0.00	0.00	100.00	0	0	5
Penicillium	6.82	0.00	93.18	3	0	41
Phaeosphaeria	60.00	0.00	40.00	9	0	6
Phanerochaete	0.00	0.00	100.00	0	0	2
Phoma	24.49	0.00	75.51	12	0	37
Pithomyces	18.18	0.00	81.82	2	0	9
Plenodomus	0.00	0.00	100.00	0	0	5
Plicaria	0.00	0.00	100.00	0	0	4
Preussia	0.00	0.00	100.00	0	0	5
Pseudopithomyces	0.00	0.00	100.00	0	0	2
Pyronema	0.00	0.00	100.00	0	0	15
Rhizopus	37.50	0.00	62.50	3	0	5
Sarocladium	0.00	0.00	100.00	0	0	7
Sclerostagonospora	42.42	0.00	57.58	14	0	19
Scopulariopsis	0.00	0.00	100.00	0	0	2
Septoria	25.00	0.00	75.00	1	0	3
Sordaria	45.45	0.00	54.55	5	0	6
Stagonosporopsis	42.86	0.00	57.14	6	0	8
Stemphylium	13.51	0.00	86.49	5	0	32
Tricharina	0.00	0.00	100.00	0	0	2
Trichoderma	0.00	0.00	100.00	0	0	2
Wallemia	0.00	0.00	100.00	0	0	2
Xenocylindrosporium	0.00	0.00	100.00	0	0	7

Note: because the primary goal of this summary is to weight the degree of significant interactions, unclassified pairings, i.e., pairings removed from the analysis by the co-occurrence (CO) threshold, were treated as random (see section 3 for further explanation).

Table S3. Percentage of total positive, negative, or random pairwise interactions for each molecular operational taxonomic unit (MOTU) and their respective number of counts.

MOTU	Percentage (%) of pairwise interactions			Positive counts	Negative counts	Random counts
	Positive	Negative	Random[1]			
Allophoma MOTU 003	0.00	0.00	100.00	0	0	3
Alternaria MOTU 006	0.00	0.00	100.00	0	0	1
Alternaria MOTU 008	0.00	0.00	100.00	0	0	5
Alternaria MOTU 009	3.49	0.58	95.93	6	1	165
Alternaria MOTU 010	0.00	0.00	100.00	0	0	3
Alternaria MOTU 011	0.00	0.00	100.00	0	0	1
Alternaria MOTU 012	0.00	0.00	100.00	0	0	3
Alternaria MOTU 013	0.00	0.00	100.00	0	0	1
Alternaria MOTU 014	20.00	0.00	80.00	1	0	4
Alternaria MOTU 015	41.51	0.00	58.49	22	0	31
Alternaria MOTU 016	0.00	0.00	100.00	0	0	1
Alternaria MOTU 017	0.00	0.00	100.00	0	0	1
Alternaria MOTU 018	0.00	0.00	100.00	0	0	1
Alternaria MOTU 019	40.00	0.00	60.00	2	0	3
Arthrinium MOTU 023	0.00	0.00	100.00	0	0	1
Arthrinium MOTU 024	0.00	0.00	100.00	0	0	1
Aspergillus MOTU 027	28.57	0.00	71.43	2	0	5
Aspergillus MOTU 028	0.00	0.00	100.00	0	0	7
Aspergillus MOTU 030	0.00	0.00	100.00	0	0	3
Aspergillus MOTU 032	0.00	0.00	100.00	0	0	1
Atrocalyx MOTU 033	0.00	0.00	100.00	0	0	1
Atrocalyx MOTU 034	0.00	0.00	100.00	0	0	3
Botrytis MOTU 039	0.00	12.50	87.50	0	1	7
Chaetomium MOTU 040	0.00	0.00	100.00	0	0	3
Chaetomium MOTU 041	0.00	0.00	100.00	0	0	7
Chaetomium MOTU 042	0.00	0.00	100.00	0	0	1
Chaetomium MOTU 043	0.00	0.00	100.00	0	0	1
Chaetomium MOTU 044	0.00	0.00	100.00	0	0	1
Cladosporium MOTU 045	20.00	0.00	80.00	1	0	4
Cladosporium MOTU 046	0.00	0.00	100.00	0	0	1
Cladosporium MOTU 048	0.00	0.00	100.00	0	0	3
Cladosporium MOTU 049	14.29	0.00	85.71	1	0	6
Cladosporium MOTU 050	0.00	0.00	100.00	0	0	1

Cladosporium MOTU 051	0.00	0.00	100.00	0	0	5
Cladosporium MOTU 053	14.29	0.00	85.71	1	0	6
Cladosporium MOTU 054	0.00	0.00	100.00	0	0	7
Cladosporium MOTU 055	6.67	0.00	93.33	1	0	14
Cladosporium MOTU 056	0.00	0.00	100.00	0	0	15
Cladosporium MOTU 057	0.00	0.00	100.00	0	0	3
Cladosporium MOTU 058	0.00	0.00	100.00	0	0	63
Cladosporium MOTU 059	6.25	0.00	93.75	2	0	30
Cladosporium MOTU 060	4.17	0.00	95.83	1	0	23
Cladosporium MOTU 061	0.00	0.00	100.00	0	0	1
Colletotrichum MOTU 062	0.00	0.00	100.00	0	0	1
Colletotrichum MOTU 065	0.00	0.00	100.00	0	0	1
Coniochaeta MOTU 066	0.00	0.00	100.00	0	0	1
Coniothyrium MOTU 067	0.00	0.00	100.00	0	0	7
Coniothyrium MOTU 068	0.00	0.00	100.00	0	0	1
Diaporthe MOTU 075	20.00	0.00	80.00	1	0	4
Diaporthe MOTU 077	28.57	0.00	71.43	2	0	5
Diaporthe MOTU 081	0.00	0.00	100.00	0	0	1
Diaporthe MOTU 082	0.00	0.00	100.00	0	0	7
Diatrypella MOTU 085	0.00	0.00	100.00	0	0	1
Didymella MOTU 086	27.27	0.00	72.73	3	0	8
Didymella MOTU 087	0.00	0.00	100.00	0	0	1
Didymella MOTU 088	0.00	0.00	100.00	0	0	3
Didymella MOTU 089	25.00	0.00	75.00	2	0	6
Didymella MOTU 090	28.57	0.00	71.43	2	0	5
Didymella MOTU 091	0.00	0.00	100.00	0	0	1
Didymella MOTU 093	0.00	0.00	100.00	0	0	1
Didymella MOTU 094	0.00	0.00	100.00	0	0	1
Didymella MOTU 096	0.00	0.00	100.00	0	0	1
Didymocyrtis MOTU 098	0.00	0.00	100.00	0	0	3
Didymocyrtis MOTU 100	0.00	0.00	100.00	0	0	1
Dimorpha MOTU 105	0.00	0.00	100.00	0	0	1
Dothiorella MOTU 107	0.00	0.00	100.00	0	0	1
Ectophoma MOTU 110	0.00	0.00	100.00	0	0	1
Epicoccum MOTU 111	23.08	0.00	76.92	3	0	10
Epicoccum MOTU 113	0.00	0.00	100.00	0	0	1
Epicoccum MOTU 114	15.38	0.00	84.62	2	0	11

Epicoccum MOTU 115	0.00	0.00	100.00	0	0	1
Epicoccum MOTU 116	0.00	0.00	100.00	0	0	1
Epicoccum MOTU 118	18.75	0.00	81.25	3	0	13
Fasciatispora MOTU 122	0.00	0.00	100.00	0	0	7
Fusarium MOTU 125	33.33	0.00	66.67	1	0	2
Fusarium MOTU 126	0.00	0.00	100.00	0	0	1
Fusarium MOTU 131	0.00	0.00	100.00	0	0	5
Fusarium MOTU 132	36.36	0.00	63.64	4	0	7
Fusarium MOTU 133	33.33	0.00	66.67	1	0	2
Fusarium MOTU 137	28.57	0.00	71.43	2	0	5
Fusarium MOTU 138	0.00	0.00	100.00	0	0	1
Fusarium MOTU 139	28.57	0.00	71.43	2	0	5
Hendersonia MOTU 144	40.00	0.00	60.00	2	0	3
Hendersonia MOTU 145	33.33	0.00	66.67	1	0	2
Keissleriella MOTU 146	0.00	0.00	100.00	0	0	3
Keissleriella MOTU 147	25.00	0.00	75.00	2	0	6
Keissleriella MOTU 148	28.57	0.00	71.43	2	0	5
Leptosphaerulina MOTU 152	0.00	0.00	100.00	0	0	1
Libertasomyces MOTU 154	25.00	0.00	75.00	2	0	6
Libertasomyces MOTU 155	33.33	0.00	66.67	1	0	2
Libertasomyces MOTU 156	0.00	0.00	100.00	0	0	1
Libertasomyces MOTU 157	0.00	0.00	100.00	0	0	1
Libertasomyces MOTU 158	8.33	0.00	91.67	2	0	22
Libertasomyces MOTU 159	0.00	0.00	100.00	0	0	1
Morinia MOTU 162	0.00	0.00	100.00	0	0	1
Morinia MOTU 163	0.00	0.00	100.00	0	0	1
Neodeightonia MOTU 164	0.00	0.00	100.00	0	0	7
Neodeightonia MOTU 165	0.00	0.00	100.00	0	0	1
Neodidymelliopsis MOTU 166	33.33	0.00	66.67	1	0	2
Neodidymelliopsis MOTU 167	0.00	0.00	100.00	0	0	1
Neodidymelliopsis MOTU 168	20.00	0.00	80.00	4	0	16
Neodidymelliopsis MOTU 169	0.00	0.00	100.00	0	0	3
Neofusicoccum MOTU 174	33.33	0.00	66.67	1	0	2
Neofusicoccum MOTU 175	0.00	0.00	100.00	0	0	1
Neofusicoccum MOTU 176	0.00	0.00	100.00	0	0	1
Neopestalotiopsis MOTU 178	0.00	0.00	100.00	0	0	1
Neosetophoma MOTU 185	20.63	0.00	79.37	13	0	50

Neosetophoma MOTU 186	0.00	0.00	100.00	0	0	1
Neosetophoma MOTU 187	31.25	0.00	68.75	5	0	11
Neosetophoma MOTU 188	39.36	1.06	59.57	37	1	56
Neosetophoma MOTU 190	33.33	0.00	66.67	1	0	2
Neosetophoma MOTU 191	0.00	0.00	100.00	0	0	1
Neosulcatispora MOTU 193	0.00	0.00	100.00	0	0	1
Nigrospora MOTU 195	0.00	0.00	100.00	0	0	1
Nigrospora MOTU 196	0.00	0.00	100.00	0	0	1
Nothodactylaria MOTU 200	0.00	0.00	100.00	0	0	3
Nothophoma MOTU 202	37.5	0.00	62.50	3	0	5
Paraconiothyrium MOTU 204	0.00	0.00	100.00	0	0	1
Paraconiothyrium MOTU 206	0.00	0.00	100.00	0	0	1
Paraconiothyrium MOTU 208	0.00	0.00	100.00	0	0	1
Parastagonospora MOTU 210	0.00	0.00	100.00	0	0	3
Penicillium MOTU 211	0.00	0.00	100.00	0	0	3
Penicillium MOTU 212	0.00	0.00	100.00	0	0	1
Penicillium MOTU 214	0.00	0.00	100.00	0	0	3
Penicillium MOTU 216	0.00	0.00	100.00	0	0	3
Penicillium MOTU 217	30.77	0.00	69.23	4	0	9
Penicillium MOTU 218	0.00	0.00	100.00	0	0	7
Penicillium MOTU 219	0.00	0.00	100.00	0	0	3
Penicillium MOTU 220	0.00	0.00	100.00	0	0	1
Penicillium MOTU 221	12.50	0.00	87.50	3	0	21
Penicillium MOTU 222	0.00	0.00	100.00	0	0	1
Phaeosphaeria MOTU 224	28.57	0.00	71.43	2	0	5
Phaeosphaeria MOTU 226	0.00	0.00	100.00	0	0	1
Phaeosphaeria MOTU 227	0.00	0.00	100.00	0	0	1
Phoma MOTU 234	0.00	0.00	100.00	0	0	3
Phoma MOTU 235	24.53	0.00	75.47	13	0	40
Phoma MOTU 239	20.00	0.00	80.00	1	0	4
Phoma MOTU 240	28.57	0.00	71.43	2	0	5
Phoma MOTU 243	6.38	0.00	93.62	6	0	88
Phoma MOTU 244	0.00	0.00	100.00	0	0	1
Phoma MOTU 245	0.00	0.00	100.00	0	0	13
Phoma MOTU 246	66.67	0.00	33.33	2	0	1
Phoma MOTU 247	28.57	0.00	71.43	2	0	5
Phoma MOTU 248	0.00	0.00	100.00	0	0	3

Phoma MOTU 249	28.57	0.00	71.43	2	0	5
Phoma MOTU 250	0.00	0.00	100.00	0	0	1
Pithomyces MOTU 254	0.00	0.00	100.00	0	0	3
Pithomyces MOTU 255	0.00	0.00	100.00	0	0	1
Plenodomus MOTU 257	0.00	0.00	100.00	0	0	1
Plenodomus MOTU 258	0.00	0.00	100.00	0	0	1
Plicaria MOTU 259	0.00	0.00	100.00	0	0	1
Preussia MOTU 263	0.00	0.00	100.00	0	0	1
Pyronema MOTU 268	9.09	0.00	90.91	1	0	10
Rhizopus MOTU 270	0.00	0.00	100.00	0	0	3
Sarocladium MOTU 274	0.00	0.00	100.00	0	0	1
Sclerostagonospora MOTU 281	25.00	0.00	75.00	2	0	6
Sclerostagonospora MOTU 282	0.00	0.00	100.00	0	0	1
Sclerostagonospora MOTU 283	25.00	0.00	75.00	2	0	6
Sclerostagonospora MOTU 284	18.18	0.00	81.82	2	0	9
Sclerostagonospora MOTU 286	0.00	0.00	100.00	0	0	1
Septoria MOTU 289	0.00	0.00	100.00	0	0	1
Sordaria MOTU 290	0.00	0.00	100.00	0	0	1
Sordaria MOTU 292	0.00	0.00	100.00	0	0	1
Stagonosporopsis MOTU 297	0.00	0.00	100.00	0	0	3
Stagonosporopsis MOTU 298	40.00	0.00	60.00	2	0	3
Stemphylium MOTU 299	0.00	0.00	100.00	0	0	1
Stemphylium MOTU 301	0.00	0.00	100.00	0	0	1
Stemphylium MOTU 303	10.00	5.00	85.00	2	1	17
Stemphylium MOTU 304	14.29	0.00	85.71	1	0	6
Stemphylium MOTU 306	25.00	0.00	75.00	2	0	6
Trichoderma MOTU 315	0.00	0.00	100.00	0	0	1
Xenocylindrosporium MOTU 319	0.00	0.00	100.00	0	0	1
Xenocylindrosporium MOTU 320	0.00	0.00	100.00	0	0	1

Note: because the primary goal of this summary is to weight the degree of significant interactions, unclassified pairings, i.e., pairings removed from the analysis by the co-occurrence (CO) threshold, were treated as random (see section 3 for further explanation).

Table S4. Significant genus pairwise interactions and their respective probabilities.

Genera in pairwise interaction		Patterns of co-occurrence (CO)[1]			Probability of CO[2]	
		Observed	Probability	Expected	Less than observed	More than observed
Alternaria	*Cladosporium*	54	0.36	47.7	0.99248	0.01915
Alternaria	*Phoma*	48	0.30	40.4	0.99810	0.00565
Cladosporium	*Phoma*	45	0.29	38.4	0.99363	0.01632
Neosetophoma	*Phoma*	33	0.18	23.6	0.99983	0.00065
Cladosporium	*Penicillium*	26	0.15	19.8	0.99717	0.00967
Alternaria	*Epicoccum*	29	0.15	19.6	1.00000	4.00×10^{-5}
Alternaria	*Stemphylium*	25	0.13	17.7	0.99980	0.00118
Cladosporium	*Stemphylium*	22	0.13	16.9	0.99282	0.02294
Epicoccum	*Phoma*	26	0.12	15.8	1.00000	3.00×10^{-5}
Alternaria	*Fusarium*	21	0.10	13.5	0.99999	0.00011
Cladosporium	*Fusarium*	17	0.10	12.8	0.98886	0.03782
Alternaria	*Didymella*	19	0.10	12.2	0.99998	0.00034
Alternaria	*Neodidymelliopsis*	19	0.09	11.6	1.00000	4.00×10^{-5}
Phoma	*Sclerostagonospora*	17	0.09	11.3	0.99799	0.00830
Alternaria	*Diaporthe*	16	0.08	11.0	0.99902	0.00702
Fusarium	*Phoma*	19	0.08	10.8	0.99999	0.00011
Alternaria	*Aspergillus*	15	0.08	10.4	0.99835	0.01102
Didymella	*Phoma*	16	0.07	9.90	0.99956	0.00261
Neodidymelliopsis	*Phoma*	15	0.07	9.40	0.99912	0.00478
Neosetophoma	*Sclerostagonospora*	18	0.06	8.20	1.00000	1.00×10^{-5}
Fusarium	*Neosetophoma*	15	0.06	7.90	0.99986	0.00079
Libertasomyces	*Neosetophoma*	15	0.06	7.90	0.99986	0.00079
Keissleriella	*Phoma*	12	0.06	7.90	0.99400	0.02572
Didymella	*Neosetophoma*	17	0.05	7.20	1.00000	0.00000
Epicoccum	*Stemphylium*	12	0.05	6.90	0.99586	0.01454
Neodidymelliopsis	*Neosetophoma*	15	0.05	6.80	1.00000	5.00×10^{-5}
Diaporthe	*Neosetophoma*	12	0.05	6.40	0.99914	0.00443
Keissleriella	*Neosetophoma*	13	0.04	5.70	0.99999	0.00011
Alternaria	*Phaeosphaeria*	9	0.04	5.50	1.00000	0.01005
Epicoccum	*Fusarium*	9	0.04	5.30	0.98710	0.04212
Alternaria	*Nothophoma*	8	0.04	4.90	1.00000	0.01711
Didymella	*Epicoccum*	11	0.04	4.80	0.99982	0.00109
Epicoccum	*Neodidymelliopsis*	10	0.03	4.50	0.99941	0.00323

Hendersonia	*Phoma*	9	0.03	4.40	1.00000	0.00127
Phaeosphaeria	*Phoma*	9	0.03	4.40	1.00000	0.00127
Aspergillus	*Penicillium*	11	0.03	4.30	0.99997	0.00027
Diaporthe	*Epicoccum*	9	0.03	4.30	0.99816	0.00884
Alternaria	*Pithomyces*	7	0.03	4.30	1.00000	0.02896
Alternaria	*Sordaria*	7	0.03	4.30	1.00000	0.02896
Nothophoma	*Phoma*	8	0.03	3.90	1.00000	0.00276
Diaporthe	*Stemphylium*	9	0.03	3.90	0.9993	0.00399
Fusarium	*Sclerostagonospora*	10	0.03	3.80	0.99992	0.00058
Libertasomyces	*Sclerostagonospora*	10	0.03	3.80	0.99992	0.00058
Fusarium	*Libertasomyces*	8	0.03	3.60	0.99775	0.01080
Alternaria	*Rhizopus*	6	0.03	3.70	1.00000	0.04878
Didymella	*Sclerostagonospora*	9	0.03	3.40	0.99979	0.00141
Didymella	*Fusarium*	11	0.03	3.30	1.00000	1.00×10^{-5}
Didymella	*Libertasomyces*	9	0.03	3.30	0.99987	0.00094
Hendersonia	*Neosetophoma*	8	0.02	3.20	0.99994	0.00117
Neosetophoma	*Phaeosphaeria*	7	0.02	3.20	0.99883	0.01040
Neodidymelliopsis	*Sclerostagonospora*	9	0.02	3.30	0.99989	0.00087
Fusarium	*Neodidymelliopsis*	12	0.02	3.10	1.00000	0.00000
Libertasomyces	*Neodidymelliopsis*	10	0.02	3.10	0.99999	7.00×10^{-5}
Diaporthe	*Sclerostagonospora*	7	0.02	3.10	0.99673	0.01591
Diaporthe	*Libertasomyces*	9	0.02	3.00	0.99996	0.00033
Diaporthe	*Fusarium*	8	0.02	3.00	0.99967	0.00231
Didymella	*Neodidymelliopsis*	16	0.02	2.80	1.00000	0.00000
Neosetophoma	*Nothophoma*	7	0.02	2.90	0.99982	0.00322
Keissleriella	*Libertasomyces*	6	0.02	2.60	0.99455	0.02622
Keissleriella	*Sclerostagonospora*	11	0.02	2.70	1.00000	0.00000
Diaporthe	*Didymella*	7	0.02	2.70	0.99893	0.00650
Neosetophoma	*Sordaria*	6	0.02	2.50	0.99944	0.00860
Diaporthe	*Neodidymelliopsis*	8	0.02	2.60	0.99992	0.00069
Didymella	*Keissleriella*	9	0.02	2.40	1.00000	4.00×10^{-5}
Keissleriella	*Neodidymelliopsis*	7	0.02	2.30	0.99976	0.00198
Epicoccum	*Phaeosphaeria*	6	0.02	2.10	0.99937	0.00597
Atrocalyx	*Neosetophoma*	5	0.02	2.10	0.99829	0.02222
Neosetophoma	*Rhizopus*	5	0.02	2.10	0.99829	0.02222
Epicoccum	*Hendersonia*	5	0.02	2.10	0.99403	0.03530
Epicoccum	*Neofusicoccum*	5	0.01	1.90	0.99759	0.01900

Epicoccum	*Stagonosporopsis*	5	0.01	1.90	0.99759	0.01900
Hendersonia	*Sclerostagonospora*	8	0.01	1.50	1.00000	0.00000
Phaeosphaeria	*Sclerostagonospora*	7	0.01	1.50	1.00000	5.00×10^{-5}
Didymocyrtis	*Sclerostagonospora*	4	0.01	1.50	0.99227	0.04663
Dothiorella	*Neosetophoma*	4	0.01	1.40	1.00000	0.01515
Epicoccum	*Rhizopus*	4	0.01	1.40	0.99701	0.02879
Fusarium	*Phaeosphaeria*	7	0.01	1.50	1.00000	4.00×10^{-5}
Pithomyces	*Stemphylium*	5	0.01	1.50	0.99961	0.00533
Didymocyrtis	*Fusarium*	5	0.01	1.50	0.99938	0.00623
Fusarium	*Hendersonia*	5	0.01	1.50	0.99938	0.00623
Arthrinium	*Penicillium*	4	0.01	1.50	0.99594	0.03605
Fusarium	*Neodeightonia*	4	0.01	1.50	0.99377	0.03987
Hendersonia	*Libertasomyces*	4	0.01	1.50	0.99377	0.03987
Libertasomyces	*Neodeightonia*	4	0.01	1.50	0.99377	0.03987
Fusarium	*Nothophoma*	6	0.01	1.30	0.99999	0.00023
Neodidymelliopsis	*Phaeosphaeria*	6	0.01	1.30	0.99999	0.00024
Fusarium	*Stagonosporopsis*	5	0.01	1.30	0.99977	0.00311
Didymella	*Hendersonia*	5	0.01	1.30	0.99966	0.00388
Didymella	*Phaeosphaeria*	5	0.01	1.30	0.99966	0.00388
Hendersonia	*Neodidymelliopsis*	4	0.01	1.30	0.99700	0.02339
Libertasomyces	*Neofusicoccum*	4	0.01	1.30	0.99689	0.02492
Libertasomyces	*Stagonosporopsis*	4	0.01	1.30	0.99689	0.02492
Didymella	*Didymocyrtis*	4	0.01	1.30	0.99612	0.02827
Nothophoma	*Sclerostagonospora*	7	0.01	1.40	1.00000	1.00×10^{-5}
Sclerostagonospora	*Stagonosporopsis*	4	0.01	1.40	0.99611	0.02934
Fusarium	*Sordaria*	4	0.01	1.10	0.99869	0.01401
Aspergillus	*Coniothyrium*	4	0.01	1.10	0.99831	0.01536
Didymella	*Nothophoma*	5	0.01	1.20	0.99988	0.00191
Didymella	*Stagonosporopsis*	5	0.01	1.20	0.99988	0.00191
Colletotrichum	*Epicoccum*	4	0.01	1.20	0.99940	0.01159
Sclerostagonospora	*Sordaria*	4	0.01	1.20	0.99835	0.01662
Didymella	*Neofusicoccum*	4	0.01	1.20	0.99809	0.01743
Diaporthe	*Phaeosphaeria*	4	0.01	1.20	0.99773	0.01910
Didymella	*Sordaria*	5	0.01	1.00	0.99997	8.00×10^{-5}
Hendersonia	*Keissleriella*	5	0.01	1.10	0.99992	0.00123
Neodidymelliopsis	*Nothophoma*	5	0.01	1.10	0.99991	0.00147
Neodidymelliopsis	*Stagonosporopsis*	5	0.01	1.10	0.99991	0.00147

Diaporthe	*Neofusicoccum*	4	0.01	1.10	0.99890	0.01162
Neodidymelliopsis	*Neofusicoccum*	4	0.01	1.10	0.99853	0.01433
Diatrypella	*Neosetophoma*	3	0.01	1.10	1.00000	0.04411
Leptosphaerulina	*Neosetophoma*	3	0.01	1.10	1.00000	0.04411
Neosetophoma	*Septoria*	3	0.01	1.10	1.00000	0.04411

[1] Observed: observed number of samples having both genera; Probability: probability that both genera occur at a sample; Expected: expected number of samples having both genera.

[2] Probability of CO: probability that the two genera would co-occur at a frequency less than or more than the observed number of CO samples if the two genera were distributed randomly (independently) of one another. According to Griffith et al. [116] these probabilities can be interpreted as *p*-values, thus indicating significance levels for negative and positive CO patterns.

Note: pairwise interactions are listed in descending order of probability of CO less than the observed number of CO.

Table S5. Significant molecular operational taxonomic unit (MOTU) pairwise interactions and their respective probabilities.

Genera in pairwise interaction		Patterns of co-occurrence (CO)[1]			Probability of CO[2]	
		Observed	Probability	Expected	Less than observed	More than observed
Neosetophoma MOTU 185	*Neosetophoma* MOTU 188	19	0.06	7.40	1.00000	0.00000
Neodidymelliopsis MOTU 168	*Neosetophoma* MOTU 188	11	0.03	3.60	1.00000	1.00×10^{-5}
Neosetophoma MOTU 187	*Neosetophoma* MOTU 188	10	0.03	3.30	1.00000	6.00×10^{-5}
Alternaria MOTU 009	*Cladosporium* MOTU 049	6	0.02	3.00	1.00000	0.01525
Alternaria MOTU 015	*Neodidymelliopsis* MOTU 168	13	0.02	2.70	1.00000	0.00000
Alternaria MOTU 009	*Alternaria* MOTU 019	5	0.02	2.50	1.00000	0.03122
Alternaria MOTU 009	*Stagonosporopsis* MOTU 298	5	0.02	2.50	1.00000	0.03122
Neosetophoma MOTU 188	*Sclerostagonospora* MOTU 284	9	0.02	2.30	1.00000	0.00000
Didymella MOTU 086	*Neosetophoma* MOTU 188	8	0.02	2.30	1.00000	6.00×10^{-5}
Fusarium MOTU 132	*Neosetophoma* MOTU 188	8	0.02	2.30	1.00000	6.00×10^{-5}
Alternaria MOTU 015	*Epicoccum* MOTU 111	9	0.01	1.90	1.00000	0.00000
Alternaria MOTU 015	*Sclerostagonospora* MOTU 284	9	0.01	1.70	1.00000	0.00000
Alternaria MOTU 015	*Didymella* MOTU 086	8	0.01	1.70	1.00000	1.00×10^{-5}
Alternaria MOTU 015	*Didymella* MOTU 089	7	0.01	1.60	1.00000	3.00×10^{-5}
Diaporthe MOTU 077	*Neosetophoma* MOTU 188	6	0.01	1.50	1.00000	0.00019
Didymella MOTU 090	*Neosetophoma* MOTU 188	6	0.01	1.50	1.00000	0.00019
Fusarium MOTU 137	*Neosetophoma* MOTU 188	6	0.01	1.50	1.00000	0.00019
Keissleriella MOTU 148	*Neosetophoma* MOTU 188	6	0.01	1.50	1.00000	0.00019
Neosetophoma MOTU 188	*Phaeosphaeria* MOTU 224	6	0.01	1.50	1.00000	0.00019
Penicillium MOTU 217	*Penicillium* MOTU 221	7	0.01	1.20	1.00000	1.00×10^{-5}

Alternaria MOTU 015	*Diaporthe* MOTU 077	6	0.01	1.20	1.00000	3.00×10^{-5}
Alternaria MOTU 015	*Fusarium* MOTU 137	6	0.01	1.20	1.00000	3.00×10^{-5}
Phoma MOTU 235	*Phoma* MOTU 249	6	0.01	1.30	1.00000	0.00012
Alternaria MOTU 014	*Neosetophoma* MOTU 188	5	0.01	1.30	1.00000	0.00083
Hendersonia MOTU 144	*Neosetophoma* MOTU 188	5	0.01	1.30	1.00000	0.00083
Neosetophoma MOTU 188	*Phoma* MOTU 239	5	0.01	1.30	1.00000	0.00083
Neosetophoma MOTU 188	*Stagonosporopsis* MOTU 298	5	0.01	1.30	1.00000	0.00083
Fusarium MOTU 125	*Neosetophoma* MOTU 188	4	0.01	1.00	1.00000	0.00361
Fusarium MOTU 133	*Neosetophoma* MOTU 188	4	0.01	1.00	1.00000	0.00361
Hendersonia MOTU 145	*Neosetophoma* MOTU 188	4	0.01	1.00	1.00000	0.00361
Libertasomyces MOTU 155	*Neosetophoma* MOTU 188	4	0.01	1.00	1.00000	0.00361
Neodidymelliopsis MOTU 166	*Neosetophoma* MOTU 188	4	0.01	1.00	1.00000	0.00361
Neofusicoccum MOTU 174	*Neosetophoma* MOTU 188	4	0.01	1.00	1.00000	0.00361
Neosetophoma MOTU 188	*Neosetophoma* MOTU 190	4	0.01	1.00	1.00000	0.00361
Neosetophoma MOTU 188	*Phoma* MOTU 246	4	0.01	1.00	1.00000	0.00361
Phoma MOTU 243	*Phoma* MOTU 246	4	0.01	1.10	1.00000	0.00575
Alternaria MOTU 015	*Neosetophoma* MOTU 188	15	0.05	6.60	0.99999	8.00×10^{-5}
Neosetophoma MOTU 185	*Phoma* MOTU 235	13	0.04	5.20	0.99999	1.00×10^{-4}
Neosetophoma MOTU 185	*Neosetophoma* MOTU 187	9	0.02	2.80	0.99999	0.00013
Epicoccum MOTU 111	*Neosetophoma* MOTU 188	8	0.02	2.50	0.99999	0.00026
Libertasomyces MOTU 154	*Neosetophoma* MOTU 188	7	0.02	2.00	0.99999	0.00027
Neosetophoma MOTU 188	*Nothophoma* MOTU 202	7	0.02	2.00	0.99999	0.00027

Alternaria MOTU 015	*Libertasomyces* MOTU 154	6	0.01	1.60	0.99997	0.00067
Alternaria MOTU 015	*Stemphylium* MOTU 306	6	0.01	1.60	0.99997	0.00067
Neodidymelliopsis MOTU 168	*Neosetophoma* MOTU 187	6	0.01	1.40	0.99997	0.00048
Alternaria MOTU 015	*Keissleriella* MOTU 148	5	0.01	1.20	0.99997	0.00102
Alternaria MOTU 015	*Phaeosphaeria* MOTU 224	5	0.01	1.20	0.99997	0.00102
Alternaria MOTU 015	*Stemphylium* MOTU 304	5	0.01	1.20	0.99997	0.00102
Fusarium MOTU 132	*Phoma* MOTU 235	6	0.01	1.60	0.99993	0.00107
Didymella MOTU 090	*Neosetophoma* MOTU 185	5	0.01	1.30	0.99993	0.00180
Epicoccum MOTU 111	*Neodidymelliopsis* MOTU 168	5	0.01	1.00	0.99993	0.00112
Aspergillus MOTU 027	*Phoma* MOTU 235	5	0.01	1.30	0.99988	0.00206
Alternaria MOTU 015	*Fusarium* MOTU 132	6	0.01	1.70	0.99986	0.00175
Alternaria MOTU 015	*Phoma* MOTU 240	5	0.01	1.40	0.99981	0.00309
Neosetophoma MOTU 187	*Phoma* MOTU 235	7	0.02	2.30	0.99975	0.00212
Didymella MOTU 089	*Neosetophoma* MOTU 188	6	0.02	2.00	0.99973	0.00346
Neosetophoma MOTU 188	*Sclerostagonospora* MOTU 283	6	0.02	2.00	0.99973	0.00346
Neosetophoma MOTU 188	*Stemphylium* MOTU 306	6	0.02	2.00	0.99973	0.00346
Hendersonia MOTU 144	*Neosetophoma* MOTU 185	4	0.01	1.10	0.99964	0.00783
Alternaria MOTU 015	*Epicoccum* MOTU 114	6	0.01	1.90	0.99961	0.00378
Aspergillus MOTU 027	*Neosetophoma* MOTU 185	5	0.01	1.50	0.99961	0.00533
Epicoccum MOTU 118	*Stemphylium* MOTU 303	5	0.01	1.40	0.99952	0.00474
Phoma MOTU 235	*Phoma* MOTU 243	13	0.05	6.80	0.99935	0.00302
Alternaria MOTU 015	*Nothophoma* MOTU 202	5	0.01	1.60	0.99933	0.00712

Phoma MOTU 235	*Phoma* MOTU 247	4	0.01	1.10	0.99933	0.00954
Neosetophoma MOTU 188	*Phoma* MOTU 235	12	0.05	6.10	0.99921	0.00364
Alternaria MOTU 009	*Epicoccum* MOTU 114	9	0.03	5.10	0.9992	0.00972
Cladosporium MOTU 045	*Neosetophoma* MOTU 188	4	0.01	1.30	0.99917	0.01472
Diaporthe MOTU 075	*Neosetophoma* MOTU 188	4	0.01	1.30	0.99917	0.01472
Libertasomyces MOTU 158	*Neosetophoma* MOTU 188	9	0.03	4.10	0.99911	0.00502
Alternaria MOTU 015	*Cladosporium* MOTU 053	4	0.01	1.20	0.99898	0.01306
Alternaria MOTU 015	*Fusarium* MOTU 139	4	0.01	1.20	0.99898	0.01306
Neosetophoma MOTU 188	*Phoma* MOTU 240	5	0.01	1.80	0.99893	0.01156
Cladosporium MOTU 060	*Pyronema* MOTU 268	4	0.01	1.10	0.99877	0.01214
Alternaria MOTU 009	*Epicoccum* MOTU 118	11	0.05	6.60	0.99871	0.00961
Keissleriella MOTU 147	*Neosetophoma* MOTU 185	5	0.01	1.70	0.99867	0.01201
Neosetophoma MOTU 185	*Sclerostagonospora* MOTU 281	5	0.01	1.70	0.99867	0.01201
Neosetophoma MOTU 185	*Sclerostagonospora* MOTU 283	5	0.01	1.70	0.99867	0.01201
Alternaria MOTU 019	*Phoma* MOTU 243	4	0.01	1.40	0.99850	0.02273
Penicillium MOTU 221	*Phoma* MOTU 235	7	0.02	2.90	0.99829	0.00961
Penicillium MOTU 217	*Phoma* MOTU 235	5	0.01	1.80	0.99765	0.01661
Didymella MOTU 086	*Neosetophoma* MOTU 185	5	0.02	1.90	0.99664	0.02282
Fusarium MOTU 132	*Neosetophoma* MOTU 185	5	0.02	1.90	0.99664	0.02282
Alternaria MOTU 015	*Neosetophoma* MOTU 187	6	0.02	2.50	0.99633	0.01990
Fusarium MOTU 139	*Neosetophoma* MOTU 188	4	0.01	1.50	0.99594	0.03605
Neosetophoma MOTU 188	*Phoma* MOTU 247	4	0.01	1.50	0.99594	0.03605

Keissleriella MOTU 147	*Phoma* MOTU 235	4	0.01	1.40	0.99519	0.03425
Nothophoma MOTU 202	*Phoma* MOTU 235	4	0.01	1.40	0.99519	0.03425
Phoma MOTU 235	*Sclerostagonospora* MOTU 281	4	0.01	1.40	0.99519	0.03425
Alternaria MOTU 009	*Stemphylium* MOTU 303	11	0.05	7.10	0.99479	0.02574
Neosetophoma MOTU 185	*Phoma* MOTU 249	4	0.01	1.50	0.99467	0.03925
Cladosporium MOTU 059	*Penicillium* MOTU 217	4	0.01	1.40	0.99456	0.03535
Penicillium MOTU 217	*Phoma* MOTU 243	6	0.02	2.80	0.99440	0.03075
Libertasomyces MOTU 158	*Neosetophoma* MOTU 185	7	0.03	3.50	0.99282	0.03025
Alternaria MOTU 015	*Cladosporium* MOTU 055	5	0.02	2.10	0.99282	0.03781
Epicoccum MOTU 118	*Phoma* MOTU 243	7	0.03	3.70	0.99089	0.03870
Penicillium MOTU 221	*Phoma* MOTU 243	8	0.03	4.50	0.98788	0.04401
Alternaria MOTU 015	*Cladosporium* MOTU 059	7	0.03	3.70	0.98779	0.04496
Alternaria MOTU 009	*Botrytis* MOTU 039	1	0.03	4.10	0.02817	0.99724
Neosetophoma MOTU 188	*Stemphylium* MOTU 303	0	0.03	3.60	0.01293	1.00000
Neosetophoma MOTU 185	*Neosetophoma* MOTU 188	19	0.06	7.40	1.00000	0.00000
Neodidymelliopsis MOTU 168	*Neosetophoma* MOTU 188	11	0.03	3.60	1.00000	1.00×10^{-5}
Neosetophoma MOTU 187	*Neosetophoma* MOTU 188	10	0.03	3.30	1.00000	6.00×10^{-5}
Alternaria MOTU 009	*Cladosporium* MOTU 049	6	0.02	3.00	1.00000	0.01525
Alternaria MOTU 015	*Neodidymelliopsis* MOTU 168	13	0.02	2.70	1.00000	0.00000
Alternaria MOTU 009	*Alternaria* MOTU 019	5	0.02	2.50	1.00000	0.03122
Alternaria MOTU 009	*Stagonosporopsis* MOTU 298	5	0.02	2.50	1.00000	0.03122
Neosetophoma MOTU 188	*Sclerostagonospora* MOTU 284	9	0.02	2.30	1.00000	0.00000

Didymella MOTU 086	*Neosetophoma* MOTU 188	8	0.02	2.30	1.00000	6.00×10^{-5}
Fusarium MOTU 132	*Neosetophoma* MOTU 188	8	0.02	2.30	1.00000	6.00×10^{-5}

[1] Observed: observed number of samples having both genera; Probability: probability that both genera occur at a sample; Expected: expected number of samples having both genera.

[2] Probability of CO: probability that the two genera would co-occur at a frequency less than or more than the observed number of CO samples if the two genera were distributed randomly (independently) of one another. According to Griffith et al. [116] these probabilities can be interpreted as *p*-values, thus indicating significance levels for negative and positive CO patterns.

Note: pairwise interactions are listed in descending order of probability of CO less than the observed number of CO.
